# Calcitriol and Tacalcitol Modulate Th17 Differentiation Through Osteopontin Receptors: Age-Dependent Insights from a Mouse Breast Cancer Model

**DOI:** 10.2147/ITT.S537852

**Published:** 2025-08-23

**Authors:** Aleksandra Strzykalska-Augustyniak, Mateusz Psurski, Honorata Zachary, Beata Filip-Psurska, Dagmara Kłopotowska, Magdalena Milczarek, Marta Świtalska, Martyna Stachowicz-Suhs, Natalia Łabędź, Aleksandra Ziemblicka, Michalina Gos, Joanna Wietrzyk

**Affiliations:** 1 Hirszfeld Institute of Immunology and Experimental Therapy, Polish Academy of Science, Wrocław, Poland

**Keywords:** 4T1, metastasis, angiogenesis, vitamin D, IL-17, IFNγ

## Abstract

**Purpose:**

Beyond its direct anticancer effects in breast cancer (BC), vitamin D_3_ (VD_3_) also modulates tumor progression and metastasis through immune mechanisms. T-helper 17 (Th17) cells may play a key role in these effects. This study investigates how VD_3_ influences Th17 differentiation in 4T1 and 67NR murine BC models.

**Methods:**

Calcitriol or tacalcitol was administered to young and aged mice bearing 4T1 or 67NR tumors. Tumor growth, angiogenesis, and metastasis were evaluated. CD4^+^ lymphocytes isolated from tumors and other tissues were analyzed by flow cytometry for IL-17 and osteopontin (OPN, *Spp1*) receptors. CD4^+^ splenocytes were separated; gene expression was assessed using qPCR, and protein levels by Western blotting, ELISA. CD3^+^CD4^+^ splenocytes were ex vivo differentiated into Th17 cells with blockade of CD29, CD51, and CD44, followed by flow cytometric analysis of IL-17 and IFNγ expression.

**Results:**

Tacalcitol increased metastasis in young mice but decreased it in aged mice with 4T1 tumors. Th17 cell levels in the lungs increased in young mice treated with tacalcitol but declined in aged counterparts. IL-17^+^ and IFNγ^+^ Th17 cells increased upon differentiation from splenocytes of treated mice. CD29 promoted IL-17 expression in tacalcitol-treated mice, while CD51 and CD44 had opposing effects. CD51 blockade reduced IFNγ^+^ Th17 cells in both treatment groups. *Spp1* expression increased in CD4^+^ lymphocytes, and OPN levels were elevated in induced Th17 cells from tacalcitol-treated young mice, suggesting a role in Th17 activation.

**Conclusion:**

CD29 stimulates IL-17 expression in response to tacalcitol, while CD51 and CD44 exert opposing effects. CD51 also mediates IFNγ expression. VD_3_-induced modulation of IL-17 and IFNγ in Th17 cells may influence their pro- or anticancer function.

## Introduction

Vitamin D_3_ (VD_3_), particularly its hormonally active form, calcitriol (1,25-dihydroxycholecalciferol), has immunomodulatory properties.^1^ Its direct effects on nearly all immune cells, including lymphocytes, monocytes, and macrophages, as well as other cells within the tumor microenvironment (eg, fibroblasts and vascular endothelial cells), have been demonstrated.^1^,^2^ Consequently, the impact of VD₃ on cancer development depends not only on its direct effects on cancer cells, extensively described in the scientific literature,^3–6^ but also on its broader effects on the immune system and the tumor microenvironment.^7–10^

While the immunosuppressive effects of VD_3_ may offer potential benefits in cancer treatment;^11^ some researchers suggest these same properties could also be detrimental.^12^,^13^ Lymphocytes express the vitamin D receptor (VDR) upon activation, while dendritic cells and macrophages express it constitutively, making VD₃ a key modulator of immune and inflammatory responses.^1^,^12^,^14^ Moreover, Th17 lymphocytes express VDR, and the proinflammatory cytokine IL-17A is modulated by VD_3_ in both mouse and human T lymphocytes. Most studies suggest that calcitriol reduces Th17 cell recruitment and IL-17 secretion via the VDR-mediated pathway.^15–20^ However, in our studies in young mice, calcitriol and its analogs (eg, tacalcitol) enhanced the lung metastatic potential of 4T1 mouse mammary gland cells,^21^ while screening revealed enhanced expression of genes associated with Th17 lymphocytes in the spleen: IL-17A (*Il17a*), RAR-related orphan receptor α (*Rora*), RAR-related orphan receptor γ (*Rorc*), IL-21 (*Il21*), IL-17 receptor E (*Il17re*), and IL-1 receptor type I (*Il1r1*).^22^ In contrast, applying the same treatment to aged, ovariectomized (OVX) mice bearing 4T1 tumors led to a temporary reduction in lung metastases^23^ and decreased *Rorc* expression.^24^

CD4^+^ splenocytes coming from young tacalcitol-treated mice and stimulated ex vivo to induce Th17 cells produced higher levels of IL-17A than those from untreated control mice, whereas the opposite effect was observed in aged OVX mice.^24^ The effect of IL-17 varies depending on the stage of tumor development. In the context of chronic cancer and inflammation, IL-17’s tumor-promoting activity—mainly through the enhancement of angiogenesis—often surpasses its anticancer functions, including the stimulation of cytotoxic T lymphocytes and other immune cells that target tumors.^25^,^26^

Current evidence indicate that osteopontin (OPN) is essential for dendritic cells to facilitate Th17 cell differentiation^27^ and IL-17 production.^28^ OPN regulates IL-17 expression through its receptors,^29^ and the *Spp1* (OPN) gene promoter region contains a vitamin D response element (VDRE).^30^ The effects of VD₃ metabolites and analogs are mediated through VDR, which, upon ligand binding, dimerizes with the retinoid X receptor (RXR) and interacts with VDREs in the target genes, thereby influencing their transcription.^31^

We hypothesized that VD₃ regulates Th17 cell differentiation through VDR’s influence on OPN, potentially affecting tumor progression. Therefore, we aimed to analyze the role of OPN receptors in the differentiation of Th17 cells harvested from murine mammary gland tumors (4T1 and 67NR) treated with calcitriol and tacalcitol.

## Materials and Methods

### Cells

Mouse mammary gland cancer cell lines (4T1 and 67NR) were cultured under standard conditions to establish orthotopic tumor models. 4T1 and 67NR cells were procured from the American Type Culture Collection (ATCC, Rockville, MD, USA) and the Barbara Ann Karmanos Cancer Institute (Detroit, MI, USA), respectively. 4T1 cells were maintained in RPMI 1640 medium (Gibco™, Thermo Fisher Scientific, Waltham, MA, USA) with 10% (v/v) fetal bovine serum (FBS; HyClone, GE Healthcare, Chicago, IL, USA), 1 mM sodium pyruvate, and 3.5 g/L glucose (both Sigma-Aldrich, St. Louis, MO, USA). The 67NR cell line was cultured in Dulbecco’s modified Eagle medium (DMEM; Gibco, Scotland, UK) containing 10% (v/v) calf bovine serum (CBS; ATCC, Rockville, MD, USA), 1% amino acids, and 2 mM L-glutamine (both Sigma-Aldrich Chemie GmbH, Steinheim, Germany). Culture media were further supplemented with 100 µg/mL streptomycin and 100 U/mL penicillin (Sigma-Aldrich Chemie GmbH, Steinheim, Germany, and Polfa Tarchomin S.A., Warsaw, Poland, respectively). All cell lines were incubated at 37 °C in a humidified atmosphere of 5% (v/v) CO_2_.

### Mice

BALB/c/Foxp3GFP mice were generated for this study by systematically interbreeding BALB/c mice (Animalab, Poznań, Poland; Hirszfeld Institute of Immunology and Experimental Therapy, Polish Academy of Sciences [HIIET PAS], Wrocław, Poland) with C57BL/6/Foxp3GFP (C57BL/6-Tg(Foxp3-GFP)90Pkraj) mice, which express green fluorescent protein (GFP) under the *Foxp3* gene promoter.^32^ Progeny were screened for Foxp3GFP reporter expression,^33^ and all animals were housed at HIIET PAS, Wrocław, Poland.

Animal experiments were performed with approval from the Local Ethics Committee for Animal Experiments, Wrocław, Poland (permissions No. 50/2020 for experimental procedures and No. 44/2019, covering transgenic animal testing during breeding). All procedures adhered to the 3R principles, Directive 2010/63/EU, and national regulations. Mice were maintained in a specific pathogen-free facility with a 12/12-h light/dark cycle and provided SAFE 132 fodder (SAFE, Rosenberg, Germany) *ad libitum*.

This investigation utilized 6- to 8-week-old and 36- to 40-week-old mice. To establish the postmenopausal model, aged mice (30–35 weeks old) underwent ovariectomy (OVX) 5–10 weeks before the start of experiments. Ovariectomy was performed under general anesthesia using 3% (v/v) isoflurane inhalation (Aerane isofluranum, Baxter, Canada) in synthetic air (200 mL/min) and a buprenorphine injection (0.2 mg/kg; Orion Pharma Poland, Warsaw, Poland), as previously described.^23^ As a control for the procedure, mice underwent sham surgery. The uterine weight confirmed successful ovariectomy (Supplementary Figure S1A). Post-surgical care included buprenorphine (0.1 mg/kg) for 24 h and as needed subsequently, with Dermatol powder (Galenic Laboratory, Olsztyn, Poland) applied to promote wound healing.^23^

### Scheme of Animal Studies

**Figure 1:**
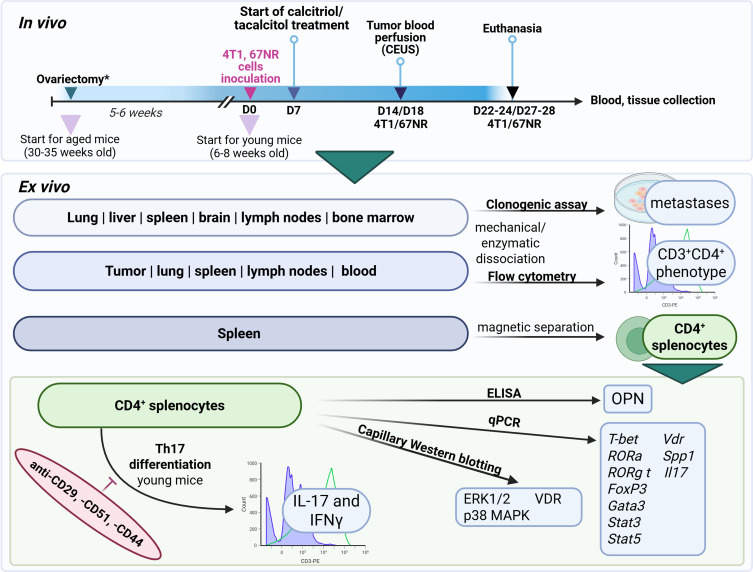
Schematic overview of the experimental design and key methodological steps. Created in BioRender. Filip-Psurska, (B) (2025) https://BioRender.com/49ip4xx.

**Note**: *Indicates aged mice.

**Abbreviations**: CEUS, contrast-enhanced ultrasonography; OPN, *Spp1*, osteopontin; VDR, vitamin D receptor; *T-bet*, Tbx21, T-Box Transcription Factor 21; *RORa*, RAR-related orphan receptor A;* RORg t*, RAR-related orphan receptor C (Rorc/ROR-γt);* FoxP3*, forkhead box P3; *Gata3*, GATA Binding Protein 3;* Stat3, Stat5*, signal transducer and activator of transcription 3; 5a, IL-17, *Il17*, interleukin 17a.

Orthotopic implantation of 4T1 (1×10^4^) or 67NR (2×10^5^) cells into the second right mammary fat pad was performed on day 0. Beginning 7 days post-tumor cell inoculation, mice received calcitriol (0.5 µg/kg) or tacalcitol (1 µg/kg) via oral gavage three times weekly. Tumor volume and body weight were monitored tri-weekly throughout the study. Tumor volume (TV) was determined using the following formula: $TV \left[{{\mathrm{m}}{{\mathrm{m}}^3}} \right] = \left({{a^2} \times b} \right)/2$, where *a* and *b* represent the shorter and longer tumor diameters, respectively, measured with a caliper. On day 14 (4T1) or day 18 (67NR), blood flow analysis was performed using contrast-enhanced ultrasonography (CEUS). Mice in the 4T1 cancer model were sacrificed on days 22–24 after cancer cell inoculation, while 67NR tumor-bearing mice were sacrificed on days 27–28. Tumors, spleens, lungs, livers, lymph nodes, brains, bone marrow, and blood were collected from all animals for further analysis (Figure 1).

For each tumor model, four independent experiments were conducted, with five mice per treatment group in each experiment. The exact number of animals or replicates used in each assay is stated in the figure legends.

### Contrast-Enhanced Ultrasonography (Tumor Blood Perfusion)

Contrast-enhanced ultrasonography (CEUS) was used to assess tumor vascularization and blood flow dynamics. The MicroMarker™ contrast agent (VisualSonics, Ontario, Canada), approximately 10^9^ particles per mouse, was reconstituted in 1 mL of sterile 0.9% saline solution. Animals were anesthetized via continuous inhalation of 2–3% isoflurane (Baxter, Deerfield, Germany) in synthetic air (200 mL/min) and secured on the treatment platform. Following application of air bubble–free gel, the tumor’s central cross-section was visualized in the transverse plane with an MS-250S scanhead (Vevo 2100 ultrasound imaging system; VisualSonics). A 100 µL bolus of the contrast agent was administered intravenously, and the initial imaging sequence (bolus phase) was recorded at approximately 15 frames per second. Once the contrast signal within the tumor stabilized (ca. 50s), microbubbles in the field of view were disrupted using burst mode, and a subsequent imaging sequence (replenishment phase) was acquired. Post-imaging, mice were maintained in a warm environment until complete recovery. Data were analyzed using Vevo LAB 1.7.1 software with the VevoCQ modality (VisualSonics).

### CD31 Immunohistochemistry (Blood Vessel Analysis)

Immunohistochemistry was performed to quantify tumor-associated blood vessels via CD31 staining. Detection of CD31 protein was performed using a primary anti-CD31 antibody (CD31 Polyclonal Antibody, series ZA4177363, ZC4226264, Thermo Fisher Scientific, Waltham, MA, USA) and a peroxidase-labeled secondary antibody (Goat anti-Rabbit IgG (H+L) Secondary Antibody, HRP, series YH375237, Thermo Fisher Scientific, Waltham, MA, USA). Microscopic evaluation of specimens was conducted to determine the number of blood vessels visible in the field of view. In manual assessment, a fully formed vessel or a group of positive cells was counted as one vessel, while single cells were excluded from the count. These analyses were performed by Sorbolab sp. z o.o., Poznań, Poland.

### Blood Mononuclear Cell Isolation and Blood Morphological and Biochemical Parameters

Blood samples from experimental mice were collected into VACUTTE tubes containing lithium heparin (Greiner Bio-One, 454089; A-Biotech, Wrocław, Poland). Plasma was obtained by centrifuging the samples at 2000×g for 15 min at 4°C and subsequently stored at −80°C for further investigations.

For mononuclear cell isolation, blood cells were suspended in Hank’s Balanced Salt Solution (HIIET PAS, Wrocław, Poland) and subjected to gradient density centrifugation (Ficoll Paque Premium 1.084; Sigma-Aldrich, St. Louis, MO, USA) at 400×g for 40 min at room temperature. The collected mononuclear cells were washed with phosphate-buffered saline (PBS; HIIET PAS, Wrocław, Poland), centrifuged, and promptly utilized for cytometric staining.

Whole blood parameters were analyzed using a Mythic 18 hematology analyzer (Cormay, Warsaw, Poland). Plasma concentrations of calcium (Ca²^+^), creatinine (CRE2), aspartate aminotransferase (AST), and alanine aminotransferase (ALT) were determined using a Cobas c111 ISE biochemistry analyzer (Roche Diagnostics, Warsaw, Poland).

### Cell Isolation for Further Analysis

Cells were isolated from various tissues by mechanical and enzymatic dissociation for downstream analyses.

Lymph nodes and spleens were mechanically dissociated by passing them through a 40 µm cell strainer into PBS (HIIET PAS, Wrocław, Poland) with 2% FBS (Sigma-Aldrich, St. Louis, MO, USA). The resultant cell suspension was centrifuged, and the supernatant discarded. For spleen samples, erythrocytes were lysed using Red Blood Cell Lysis Buffer (Sigma-Aldrich, St. Louis, MO, USA) at a 1:1 ratio for 1 min, followed by centrifugation and resuspension in PBS. The pellet containing separated cells was resuspended in PBS + 2% FBS and counted. The entire pellet of lymph node–isolated cells and a portion of spleen-derived cells were resuspended in 90% FBS + 10% DMSO; Tocris Bioscience, Bristol, UK) and frozen at −80°C. All steps were performed on ice using chilled reagents.

Tumor, brain, lung, and liver tissues were dissected using a scalpel and suspended in IMDM; Thermo Fisher Scientific, Waltham, MA, USA). DNase (Roche, Basel, Switzerland) and collagenase IA (*Clostridium histolyticum* collagenase; Sigma-Aldrich, St. Louis, MO, USA), both at a concentration of 1 mg/mL were added to the suspension and the mixture was incubated with shaking for 1 h at 37°C. Following enzymatic digestion, the suspension was filtered through a 40 µm cell strainer and centrifuged; the supernatant was subsequently removed. The cell pellet was resuspended in PBS containing 2% FBS and enumerated.

Remaining undigested tissue samples were placed in tubes with a ceramic homogenization bead (MP Biomedicals, Santa Ana, CA, USA) along with RIPA buffer containing protease and phosphatase inhibitor cocktails 2 and 3 (Sigma-Aldrich, St. Louis, MO, USA). Mechanical homogenization was performed using a FastPrep-24 Instrument homogenizer (5 m/s for 30s; MP Biomedicals, Santa Ana, CA, USA). Samples were incubated on ice for 30 minutes, flash-frozen in liquid nitrogen for 1 minute, and stored at −80°C. Except for the 37°C incubation, all procedures were conducted on ice using pre-chilled reagents.

Bone marrow: Femurs and tibiae were harvested from mice on ice in PBS containing antibiotics (100 U/mL penicillin, 100 µg/mL streptomycin; Polfa Tarchomin S.A., Warsaw, Poland, and Sigma-Aldrich Chemie GmbH, Steinheim, Germany, respectively). Bones were rinsed in PBS and cleaned of muscle tissue using a scalpel. After removing the epiphyses, the bone marrow was flushed out with PBS + 2% FBS. The collected suspension was centrifuged, and the supernatant was removed, followed by washing with PBS, centrifugation, and supernatant removal. The pellet containing separated cells was either resuspended in PBS + 2% FBS for immediate use or in 90% FBS + 10% DMSO and frozen in liquid nitrogen for storage.

### Clonogenic Assay for Metastasis Identification

This assay was used to quantify metastatic cells within the lungs, liver, bone marrow, brain, spleen, and lymph nodes. Cells procured from these organs were propagated in medium appropriate for the specific tumor cell line initially implanted in the mice. Aliquots were seeded onto Ø 100 mm dishes with 10 mL of medium at the following densities: bone marrow (10×10^6^ cells); lymph nodes (0.5×10^6^ cells); lungs (0.25×10^6^ cells); liver (4.5×10^6^ cells); and spleen (5×10^6^ cells). For cultures derived from 4T1 tumor-bearing animals, 6-thioguanine (Sigma-Aldrich, St. Louis, MO, USA) was introduced 24 hours post-plating to a final concentration of 1 µM/mL. Media were refreshed one to two times weekly, contingent upon cellular proliferation rates. Incubation periods were tissue-specific: 3 weeks for preparations from lungs, spleen, liver, and lymph nodes, and 2 weeks for those from spleen and bone marrow. Material from identical tissue types, obtained from three murine groups matched for age and inoculated tumor cell line, was processed for equivalent durations.

After incubation, plates were rinsed with PBS, and colonies were stained using a 1% crystal violet solution in 80% methanol (Avantor, Gliwice, Poland) for 30 minutes at room temperature. Subsequently, plates were washed with distilled water and air-dried at room temperature for 24 hours. Images of the stained colonies were captured with the ChemiDoc Imaging System (BioRad, Hercules, CA, USA). The percentage of dish area covered by colonies was quantified using ImageJ 1.53q software (Wayne Rasband and contributors, National Institutes of Health, USA).

### Magnetic Separation of CD4^+^ Splenocytes

CD4^+^ lymphocytes were isolated from mouse spleens using a magnetic separation kit (Miltenyi Biotec, Auburn, CA, USA). Splenocytes were centrifuged at 300×*g* for 7 min at 4°C and counted. Subsequently, the cells were resuspended in separation buffer (PBS with 0.5% bovine serum albumin (BSA) and 2 mM EDTA; pH 7.2; HIIET PAS, Wrocław, Poland) and incubated with an anti-CD16/CD32 blocking antibody (TruStain FcX, BioLegend, San Diego, CA, USA) for 5 min at 4°C to prevent non-specific Fc receptor binding. This step was omitted for cells designated for Th17 differentiation.

Magnetic separation proceeded according to the manufacturer’s instructions. Briefly, anti-CD4 magnetic beads (L3T4) MicroBeads (Miltenyi Biotec, Auburn, CA, USA) were added to the cell pellet, followed by a 30-minute incubation at 4°C. Post-incubation, cells were washed with separation buffer and centrifuged at 300×g for 7 minutes at 4°C. After aspirating the supernatant, the cell pellet was resuspended in fresh separation buffer. MS columns (Miltenyi Biotec, Auburn, CA, USA) were placed in a magnetic stand and equilibrated with separation buffer. The cell suspension was then applied to the column. After the flow-through was collected, the column was washed twice with separation buffer. To elute the magnetically retained CD4^+^ T cells, the column was removed from the magnetic field, and 1 mL of separation buffer was applied. The eluted cells were then counted and utilized for subsequent analyses.

### Ex vivo Th17 Differentiation Assay

Th17 cells were generated ex vivo by differentiating CD4^+^ splenocytes isolated from young mice bearing 4T1 tumors. Initially, 24-well plates were coated with anti-CD3 and anti-CD28 antibodies (both 5 µg/mL; BioLegend, San Diego, CA, USA) in sterile PBS (500 µL/well) and overnight incubated at 4°C. Next, after washing plates twice with PBS, 0.5×10^6^ CD4^+^ splenocytes were seeded per well. The culture medium consisted of IMDM supplemented with GlutaMax and β-mercaptoethanol (5×10^−5^ M) (both Thermo Fisher Scientific, Waltham, MA, USA), 10% FBS (HyClone, GE Healthcare, Chicago, IL, USA), 100 µg/mL streptomycin, 100 U/mL penicillin, IL-6 (50 ng/mL), and TGF-β (1 ng/mL) (both BioLegend, San Diego, CA, USA). These cells were cultured for 4 days under conditions of 5% CO_2_, 37°C, and 95% humidity.

After the 4-day differentiation, Th17 lymphocytes were collected for further analysis, and the corresponding supernatants were stored at −80°C for subsequent investigations. For flow cytometry, a subset of differentiated Th17 cells was additionally incubated with PMA (50 ng/mL), ionomycin (500 ng/mL), and brefeldin A (5 µg/mL) for 4 hours under 5% CO_2_, 37°C, and 95% humidity.

### Osteopontin Receptor Blocking During Th17 Differentiation

Blocking antibodies were used to study the role of OPN receptors (CD29, CD51, CD44) in Th17 cell differentiation. After CD4^+^ splenocyte separation from young mice bearing 4T1 tumors, cells were incubated with blocking antibodies targeting specific proteins (Table 1) in IMDM medium containing GlutaMax (Thermo Fisher Scientific, Waltham, MA, USA), 10% FBS; HyClone, GE Healthcare, Chicago, IL, USA), 100 U/mL penicillin, 100 µg/mL streptomycin, and β-mercaptoethanol (5 × 10^−5^ M, Thermo Fisher Scientific, Waltham, MA, USA). Incubation was performed for 30 min in uncoated 24-well plates under conditions of 5% CO_2_, 37°C, and 95% humidity. After incubation, the cells and medium were collected, supplemented with differentiation factors (IL-6 at 50 ng/mL and TGF-β at 1 ng/mL; BioLegend, San Diego, CA, USA), and transferred to plates precoated with anti-CD3 and anti-CD28 antibodies (both 5 µg/mL, BioLegend, San Diego, CA, USA). The cells were then cultured for 4 days. At the final step of the experiment, PMA (50 ng/mL), ionomycin (500 ng/mL), and brefeldin A (5 µg/mL; all from Sigma-Aldrich, St. Louis, MO, USA) were introduced to each well for a final 4-hour stimulation. After completing the differentiation process, induced Th17 lymphocytes were analyzed using flow cytometry to assess intracellular IL-17 and IFNγ levels.

**Table 1:** Antibodies are Used to Block Osteopontin Receptors

Antigen	Antibody Class (Clone), Catalog No	Concentration	Manufacturer
CD44 Monoclonal Antibody	IgG2b, kappa (IM7), catalog no 14–0441-85	2 mg/mL	eBioscience, San Diego, CA, USA
Rat IgG2b kappa Isotype Control	IgG2b, kappa (eB149/10H5), catalog no 16–4031-85	2 mg/mL	
CD51 (Integrin alpha V) Monoclonal Antibody	IgG1, kappa (RMV-7), catalog no 14–0512-85	2 mg/mL	
Rat IgG1 kappa Isotype Control	IgG1, kappa (eBRG1), catalog no 16–4301-85	2 mg/mL	
CD29 (Integrin beta 1) Monoclonal Antibody	IgG (eBioHMb1-1 (HMb1-1)), catalog no 16–0291-85	4 mg/mL	
Armenian Hamster IgG Isotype Control	IgG (eBio299Arm), catalog no 16–4888-85	4 mg/mL	

### Flow Cytometry (Extracellular and Intracellular Staining)

Flow cytometry was employed to quantify immune cell subsets and intracellular cytokine expression in isolated cells. Extracellular markers (CD3, CD4, CD25, CD29, CD44, CD51, CD61) and isotype controls were stained in specimens from each mouse using specific antibodies (Table 2). Cell viability was assessed with Fixable Viability Dye eFluor™ 780 (Invitrogen, Waltham, MA, USA), and non-specific antibody binding was prevented using TruStain FcX™ (anti-mouse CD16/32) (BioLegend, San Diego, CA, USA) to block Fc receptors.

**Table 2:** List of Antibodies and Fluorochromes Used in Flow Cytometry Analyses

Antigen	Fluorochrome	Ab Type and Catalog Number	Manufacturer
CD3	PE/Dazzle 594	Clone 145–2C11; hamster; catalog no 100348	BioLegend, San Diego, CA, USA
		Isotype control; clone HTK888, hamster; catalog no 400952	
CD4	Alexa Fluor 700	Clone GK1.5, rat; catalog no 100430	
		Isotype control; clone RTK4530, rat; catalog no 400628	
CD29	PerCP/Cyanine 5.5	Clone HMβ1-1, hamster; catalog no 102228	
		Isotype control; clone HTK888, hamster; catalog no 400932	
CD44	APC	Clone IM7, rat; catalog no 103012	
		Isotype control; clone RTK4530, rat; catalog no 400612	
CD51	Brilliant Violet 786	Clone RMV-7, rat; catalog no 740946	BD Biosciences, San Jose, CA, USA
		Isotype control; clone R3-34, rat; catalog no 563847	
CD61	PE/Cyanine7	Clone HMβ3-1, hamster; catalog no 104318	BioLegend, San Diego, CA, USA
		Isotype control; clone HTK888, hamster; catalog no 400922	
CD16/32	–	Clone 93, rat; catalog no 101320	
IL-17A	Brilliant Violet 421™	Clone TC11-18H10.1, rat; catalog no 506926	
		Isotype control; clone RTK2071, rat; catalog no 400430	
IFNγ	PE	Clone XMG1.2, rat; catalog no 12–7311-82	Invitrogen, Waltham, MA, USA
		Isotype control; clone eBRG1, rat; catalog no 12–4301-82	

Staining for extracellular markers: 1×10^5^ cells, initially suspended in PBS, were pelleted by centrifugation (350×g, 7 min, 4°C). The cell pellet was then suspended in 100 µL of Fixable Viability Dye eFluor™ 780 solutions in PBS + 2% FBS and incubated for 30 min in darkness at 4°C. After incubation, 700 µL PBS was added to each tube and centrifuged for 7 min, 350×*g*, at 4°C. Then, the cells were incubated in the dark for 5 min at 4°C with 50 µL of TruStain FcX™ blocking antibody. Subsequently, without an intervening centrifugation step, 50 µL of the appropriate extracellular staining antibody solution (in PBS + 2% FBS) was added, and samples were incubated for 30 minutes in darkness at 4°C. Following a final wash with 700 µL PBS and centrifugation, the cell pellet was resuspended in 200 µL of PBS + 2% FBS. Samples were analyzed using an LSR Fortessa flow cytometer (BD Biosciences, San Jose, CA, USA), with data compensation and analysis performed using FACSDiva software.

Intracellular staining: Cells derived from the same tissues were stimulated to produce cytokines. A total of 5×10^5^ cells were suspended in a stimulation medium (IMDM + GlutaMax + 10% FBS + brefeldin A (5 µg/mL) + PMA (50 ng/mL) + ionomycin (500 ng/mL)) and incubated for 4 h under 5% CO_2_, 37°C, and 95% humidity. For staining, 1 × 10^5^ cells were resuspended in 100 µL of Fixable Viability Dye eFluor™ 780 solution prepared in PBS with 2% FBS and incubated in the dark for 30 min at 4°C. Cells were subsequently fixed by adding 500 µL of Fixation Buffer (BioLegend, San Diego, CA, USA) and incubating for 20 minutes in darkness at room temperature. Permeabilization was achieved by washing the cells three times with 500 µL of 1× Intracellular Staining Perm Wash Buffer (BioLegend, San Diego, CA, USA), centrifuging at 350×g for 7 minutes at room temperature after each wash. Next, 100 µL of TruStain FcX™ blocking antibody in 1× Intracellular Staining Perm Wash Buffer was applied for a 5-minute incubation in darkness at 4°C. The permeabilized cells were stained with antibodies against CD3, CD4, IL-17A, and IFNγ (Table 2), diluted in 100 µL of permeabilization buffer, and incubated at room temperature for 30 minutes in darkness. Post-incubation, cells were centrifuged, resuspended in PBS with 2% FBS, and analyzed on an LSR Fortessa flow cytometer (BD Biosciences, San Jose, CA, USA). FACSDiva software was used for data processing.

### Quantitative PCR (qPCR) for Gene Expression

Gene expression in CD4^+^ lymphocytes was analyzed using quantitative real-time PCR (qPCR). RNA was isolated from CD4^+^ splenocyte lysates—previously stimulated with PMA and ionomycin and suspended in TRI-Reagent—using the Direct-zol™ RNA Miniprep Kit (ZYMO RESEARCH, Tustin, CA, USA), in accordance with the manufacturer’s instructions. DNase digestion was performed directly on the isolation columns during the procedure. RNA concentration was measured using a NanoDrop 2000 spectrophotometer (Thermo Fisher Scientific, Waltham, MA, USA). Reverse transcription to cDNA was performed with the SuperScript IV VILO Master Mix Kit (Thermo Fisher Scientific, Waltham, MA, USA) in a Veritii 9902 thermal cycler (Life Technologies, Carlsbad, CA, USA), employing the following thermal profile: 10 min at 25°C, 10 min at 50°C, and 5 min at 85°C. Subsequent gene expression analysis utilized customized TaqMan Array 96-Well FAST Plate Mouse FoxP3 plates, pre-coated with probes for specific target genes (Table 3).

**Table 3:** List of Probes Used in the Real-Time PCR Reaction

Gene		Probe ID Number
*T-bet*	Tbx21, T-Box Transcription Factor 21	Mm00450960_m1
*RORa*	RAR-related orphan receptor A	Mm01173766_m1
*RORg t*	RAR-related orphan receptor C (Rorc/ROR-γt)	Mm01261022_m1
*FoxP3*	Forkhead box P3	Mm00475165_m1
*Gata3*	GATA Binding Protein 3	Mm01337569_m1
*Stat3*	Signal transducer and activator of transcription 3	Mm01219775_m1
*Stat5*	Signal transducer and activator of transcription 5a	Mm00839861_m1
*Vdr*	Vitamin D receptor	Mm00437297_m1
*Spp1*	Osteopontin	Mm00436767_m1
*Il17*	Interleukin 17a	Mm00439619_m1
Gapdh*	Glyceraldehyde-3-phosphate dehydrogenase	Mm99999915_g1
Actb*	Actin, beta	Mm01205647_g1
Sdha*	Succinate dehydrogenase complex flavoprotein subunit A	Mm01352366_m1
B2m*	Beta-2 microglobulin	Mm00437762_m1

**Note**: *Endogenous controls.

For gene expression analysis, 18.5 ng of cDNA (4T1 model) or 10 ng of cDNA (67NR model) per reaction was used. Each reaction mixture contained the specified amount of cDNA, 2× TaqMan™ Gene Expression Master Mix (Thermo Fisher Scientific, Waltham, MA, USA), and RNase-free DEPC-treated water. The reactions were carried out using the ViiA™ 7 device (Thermo Fisher Scientific, Waltham, MA, USA) with the cycling conditions: 40 cycles of 95°C for 15s and 60°C for 60s. Expression data were normalized using endogenous controls (*B2m, Actb*, and *Sdha* for 4T1; *B2m* and *Actb* for 67NR), and relative quantification (RQ) was determined by the ΔΔCt method, with samples from the control group serving as calibrators. Data analysis was performed using ExpressionSuite Software v1.3 (Thermo Fisher Scientific, Waltham, MA, USA).

### Capillary Western Blot (Jess Simple Western)

Capillary Western blotting (Jess system) was used to quantify protein expression in CD4^+^ T cells. Protein concentration in isolated CD4^+^ splenocytes (after incubation with PMA and ionomycin) cell lysates was measured using the Bio-Rad DC Protein Assay Kit (Bio-Rad, Hercules, CA, USA). Samples were initially centrifuged (10,000×g, 10 min, 4°C), and the supernatant was transferred to a fresh tube. Concentration of lysates was performed using Amicon Ultra-0.5 mL Centrifugal Filter Unit columns (Sigma-Aldrich, St. Louis, MO, USA). Following a subsequent centrifugation (14,000×g, 15 min, 4°C), the resulting supernatant was collected. Protein levels were quantified against a standard curve generated with bovine serum albumin (BSA; 2–0.125 mg/mL; Bio-Rad, Hercules, CA, USA) using the Lowry method. Absorbance was measured on a Synergy H4 Hybrid Multi-Mode Microplate Reader (BioTek Instruments, Inc., Winooski, VT, USA).

Samples were prepared in 0.1× Sample Buffer (ProteinSimple, Bio-Techne, Minneapolis, MN, USA), and 4× Master Mix (EZ Standard Pack 1 or 3; ProteinSimple, Bio-Techne, Minneapolis, MN, USA) was added at a 4:1 ratio. The total protein amounts used for each assay were as follows: ERK/p-ERK (1.12 µg), p38/p-p38 (1.25 µg), and VDR (3.0 µg). Prepared samples were incubated at 95°C for 5 min using a heating block and then cooled on ice. Primary antibodies (Table 4) were prepared using a milk-free antibody diluent (ProteinSimple, Bio-Techne, Minneapolis, MN, USA) at the specified dilutions. For multiplex assays testing two proteins in the near-infrared (NIR) fluorescence and chemiluminescence (CHEMI) detection channels simultaneously, secondary antibodies were diluted 1:1000 (eg, AntiMouse NIR Detection Antibody/AntiRabbit NIR Detection Antibody and AntiMouse HRP Secondary Antibody/AntiRabbit HRP Secondary Antibody; ProteinSimple, Bio-Techne, Minneapolis, MN, USA).

**Table 4:** Antibodies Used for Immunodetection Using Jess Simple Western Method

Protein	Antibody Class	Dilution and Catalog Number	Detection Channel	Manufacturer
Human/Mouse/Rat ERK1/ERK2	IgG	1:25, catalog no: MAB1018	NIR	R&D Systems, Minneapolis, MN, USA
Human/Mouse/Rat Phospho-ERK1 (T202/Y204)/ERK2 (T185/Y187)	IgG, # 269434	1:25, catalog no: MAB1576	CHEMI	
p38 MAPK (D13E1)	IgG	1:125, catalog no: 8690S	NIR	Cell Signaling Technology, Danvers, MA, USA
Phospho-p38 MAPK (Thr180/Tyr182) (28B10)	IgG1	1:125, catalog no: 9216S	CHEMI	
Vitamin D_3_ Receptor (D2K6W)	IgG	1:125, catalog no: 12550S	CHEMI	

**Notes**: Near-infrared (NIR) fluorescence and chemiluminescence (CHEMI) detection channels.

To normalize the obtained results to the total protein level, the RePlex solution and Total Protein Detection solution (ProteinSimple, Bio-Techne, Minneapolis, MN, USA) were used following the manufacturer’s protocol. Additionally, luminol-S and peroxidase solutions (ProteinSimple, Bio-Techne, Minneapolis, MN, USA) were mixed at a 1:1 ratio. All solutions were applied to a 25-capillary plate (12–230 or 66–440 kDa separation modules; ProteinSimple, Bio-Techne, Minneapolis, MN, USA) designed for Jess Simple Western analysis. The analysis was performed using the Jess Simple Western System (ProteinSimple, Bio-Techne, Minneapolis, MN, USA). Data processing was conducted using Compass software for Simple Western analysis.

### ELISA (Proteins and 25(OH)D_3_ Quantification)

The levels of selected molecules were analyzed using the ELISA method in various samples, including plasma from 4T1- or 67NR-tumor-bearing mice to determine VEGF and 25(OH)D_3_ levels, as well as supernatants from stimulated CD4^+^ splenocyte cultures and cultures of differentiated Th17 lymphocytes derived from the spleens of young, 4T1 tumor-bearing mice to determine OPN levels. Each ELISA assay was performed according to the respective manufacturer’s protocols (Table 5). Analyte concentrations in the samples were calculated from standard curves generated using CurveExpert 1.4 software.

**Table 5:** List of ELISA Kits Used in the Study

Name	Catalog Number	Tested Material	Manufacturer
OPN	DY441	Plasma	R&D Systems, Minneapolis, MN, USA
		Supernatants from cultures of stimulated CD4+ cells	
		Supernatants from cultures of differentiated Th17 lymphocytes	
VEGF	BMS619-2	Plasma	Invitrogen, Waltham, MA, USA
25(OH)D_3_	MBS2601506	Plasma	MyBioSource, San Diego, CA, USA

**Abbreviations**: OPN, osteopontin; VEGF, vascular endothelial growth factor.

### Statistical Analysis

All statistical analyses were conducted using GraphPad Prism version 7.1. Data distribution normality was evaluated using the Shapiro–Wilk test. Specific tests used: One-Way ANOVA with post hoc correction for normally distributed data, and Kruskal–Wallis test with correction for non-normal distributions. The specific statistical post hoc tests employed for individual data analyses are detailed in the corresponding figure legends. Inter-group differences were considered statistically significant when the *p*-value was less than 0.05.

## Results

### Metastasis of Mouse BC Cells to Lung, Liver, Spleen, Brain and Bone Marrow

**Figure 2:**
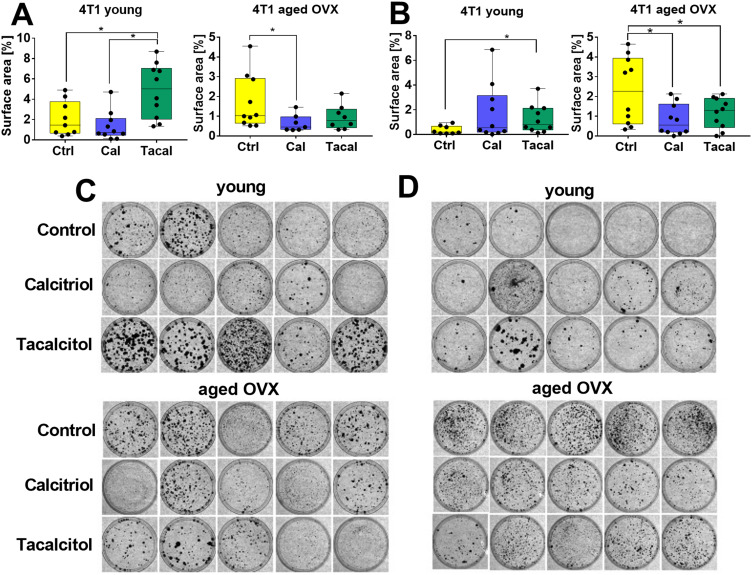
Metastasis of 4T1 mouse mammary gland tumors to lungs and liver. Surface area of 4T1 colonies cultured from (**A**) lungs and (**B**) livers harvested from 4T1 tumor-bearing young and aged OVX mice. Representative images of stained colonies: (**C**) lung and (**D**) liver. Cells isolated from organs were cultured in a medium appropriate to the tumor cell line inoculated into the mice. The percentage of the dish area covered by colonies was analyzed using the ImageJ 1.53q program. *N* = 7–10. Statistical analysis: Dunn’s test except (**B**) aged OVX: Holm-Sidak’s multiple comparison test; *p < 0.05.

**Abbreviations**: Ctrl, control; Cal, calcitriol; Tacal, tacalcitol; OVX, ovariectomized.

The results of clonogenic assay have shown that tacalcitol increased metastases to the lung and liver in young 4T1 tumor-bearing mice. In aged mice, calcitriol reduced lung metastases, while both calcitriol and tacalcitol decreased liver metastases (Figure 2A–D). 4T1 cells were also detected in the brains of both young and aged mice, in the bone marrow of young mice, and in the spleens of aged mice, without significant effects of treatment (Supplementary Figure S2A–C). The growth of 67NR cells was observed in bone marrow cultures from both young and aged mice and in lymph node cultures from young mice. Treatment did not affect the growth of these cells (Supplementary Figure S2D and E).

### Characteristics of 4T1 and 67NR Tumor Progression Upon Calcitriol and Tacalcitol Treatment

To investigate the effects of calcitriol and tacalcitol on breast cancer progression, we assessed tumor growth, vascularization, and potential toxicity of treatment using caliper measurements, contrast-enhanced ultrasonography, CD31 immunohistochemistry, blood morphology, plasma biochemistry, and ELISA assay.

Treatment with calcitriol and tacalcitol did not affect 4T1 or 67NR tumor growth in either young or aged OVX mice. The body weight of mice decreases significantly only in 4T1 and transiently in 67NR tumor-bearing young mice treated with calcitriol (Supplementary Figure S1C–F).

To further assess the toxicity of VD_3_ compounds, blood morphological and biochemical parameters were analyzed. 4T1 tumor growth induces leukocytosis,^34^ a phenomenon confirmed in this study in both young and aged mice, with increased leukocytes, including lymphocytes, monocytes, and granulocytes, compared to healthy young or sham-operated aged mice. In young mice, tacalcitol further increased the number of these cells. 67NR tumors caused a significant but less pronounced increase in white blood cell count. The number of erythrocytes and platelets, as well as hemoglobin levels, remained unchanged (Supplementary Table S1). Calcitriol, but not tacalcitol, increased Ca^2+^ plasma level. The level of 25(OH)D_3_ did not change significantly upon tumor growth and treatment. Calcitriol increased creatinine plasma levels in 4T1-bearing aged mice and 67NR-bearing young mice. Calcitriol and tacalcitol did not influence the plasma level of alanine transaminase (ALT) nor aspartate aminotransferase (AST). The level of ALT was decreased in tumor-bearing mice (Supplementary Table S2).

Time-intensity curve (TIC) parameters reflecting blood flow dynamics in tumor tissue were analyzed. These included: peak enhancement (PE), indicating the maximum intensity on the TIC and representing blood volume; time to peak (TTP), defined as the interval from baseline to maximum intensity; mean transit time (mTT), corresponding to the center of gravity of the best-fit function of echo power (or fitted signal); wash-in area under the TIC curve (WiAUC); wash-in rate (WiR), maximum slope, which reflects the rate of blood inflow; and the wash-in perfusion index (WiPI), calculated as WiAUC divided by rise time (RT, the time from the onset of enhancement to PE), serving as a measure of overall blood flow.

**Figure 3:**
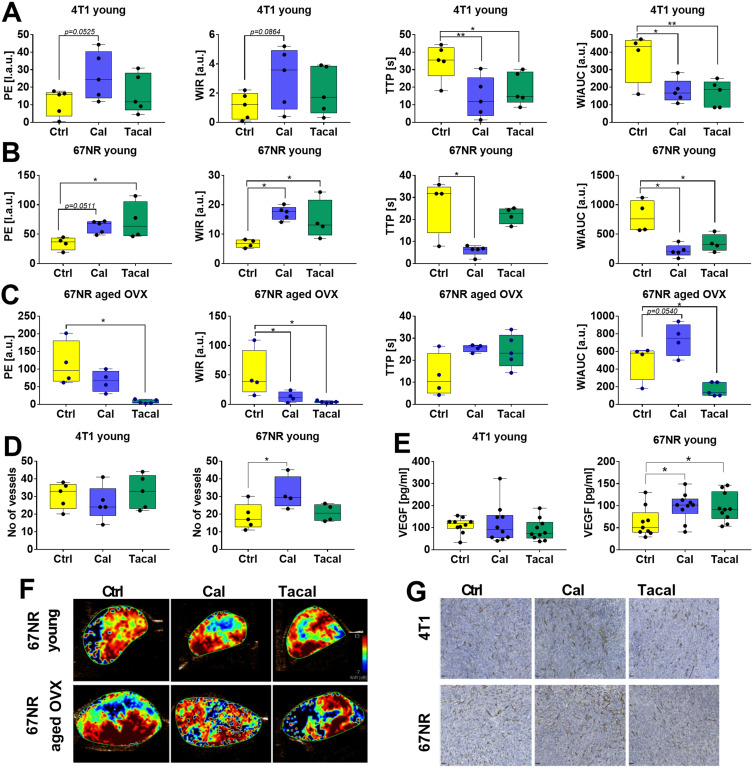
Tumor angiogenesis parameters measured in young and aged OVX mice bearing 4T1 or 67NR mouse mammary gland cancer. (**A**–**C**) contrast-enhanced ultrasonography (CEUS) analysis of blood flow after contrast agent injection in (**A**) 4T1 bearing young mice, (**B**) 67NR bearing young mice, and (**C**) 67NR bearing aged OVX mice. *N* = 4–5 mice. Parameters describing blood flow in tumor tissue: PE—peak enhancement; WiR—wash-in-rate; TTP—time to peak, WiAUC—wash-in area under the curve. (**D**) Immunohistochemical examination of tumor CD31 expression in young mice bearing 4T1 and 67NR tumors. *N* = 4–5 mice. (**E**) Vascular endothelial growth factor (VEGF) plasma level in young mice bearing 4T1 and 67NR tumors; ELISA assay. *N* = 9–10 mice. (**F**) Representative pictures of WIR parameter measured in young and aged ovariectomized mice bearing 67NR tumors. (**G**) Representative images of CD31 tumor tissue staining. Scale bars = 50 µm. Statistical analysis: (**A**–**C** and **E**) Sidak’s, (**D**) Dunnett’s multiple comparison tests; *p < 0.05; **p < 0.01.

**Abbreviations**: Ctrl, control; Cal, calcitriol; Tacal, tacalcitol; VEGF, vascular endothelial growth factor; OVX, ovariectomized.

Treatment of young mice bearing 4T1 or 67NR tumors led to a similar pattern in TIC parameters. An increase in WiR and PE indicated enhanced blood inflow into tumor tissue (Figure 3A, B and Supplementary Figure S3). A lower TTP compared to controls suggested a longer time required to reach maximal inflow intensity (Figure 3A and B). Conversely, mTT was reduced in both treatment groups in 67NR tumor-bearing mice, whereas tacalcitol increased this parameter in 4T1 tumors, suggesting either a slower or faster reperfusion process (blood inflow from adjacent tissue), respectively (Supplementary Figure S3A and C). In aged OVX mice bearing 67NR tumors, most TIC parameters showed opposite effects compared to young mice (Figure 3C and Supplementary Figure S3D). In aged mice bearing 4T1 tumors, treatments did not significantly affect blood flow parameters (Supplementary Figure S3B).

Histopathological analysis of blood vessels (CD31 staining) showed an increased number of blood vessels in young mice bearing 67NR tumors (Figure 3D–G). In 4T1 tumors, both in young and aged mice, as well as in 67NR tumors growing in aged mice, treatments did not affect CD31 expression (Figure 3D–G and Supplementary Figure S3E). Plasma VEGF concentration was elevated in calcitriol- and tacalcitol-treated young mice bearing 67NR tumors (Figure 3E) but remained unchanged in aged mice and in both young and aged 4T1 tumor-bearing mice (Figure 3E and Supplementary Figure S3F).

### IL-17 Expression in CD3^+^CD4^+^ Lymphocytes

CD4^+^ T cell subsets—including IL-17^+^ (Th17)—were characterized by flow cytometry in various tissues.

To characterize CD3^+^CD4^+^ lymphocytes, these cells were isolated from the lungs, tumor, and spleen of all mice, as well as from the blood and lymph nodes of 4T1 tumor-bearing mice. Supplementary Figure S4E–H summarizes the number of CD3^+^CD4^+^ lymphocytes in all treated mice. Significant differences in the percentage of these cells were observed in tumor tissue and lungs of aged OVX 4T1 tumor-bearing mice: tacalcitol increased CD3^+^CD4^+^ lymphocytes in both tissues, whereas calcitriol increased them only in the lungs (Supplementary Figure S4G).

**Figure 4:**
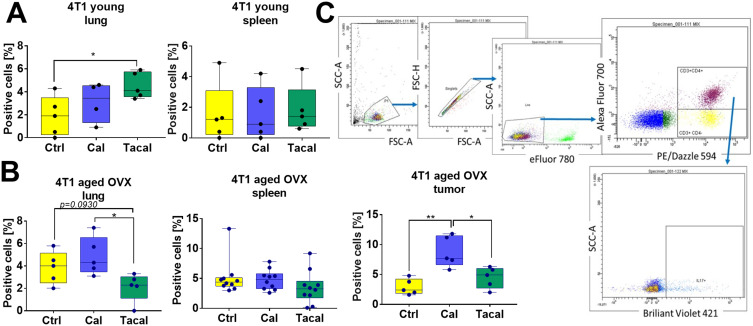
IL-17 expression (Th17 lymphocytes) in CD3^+^CD4^+^ lymphocytes isolated from 4T1 tumor-bearing mice treated with calcitriol and tacalcitol. Flow cytometry analysis of cells from (**A**) young and (**B**) aged OVX mice bearing 4T1 mouse mammary gland tumors. (**C**) Scheme of gating. *N* = 4–5 except spleens from aged OVX mice *N* = 10. Statistical analysis: (**A**) Dunn’s and (**B**) Dunnett’s multiple comparison tests; *p < 0.05, **p < 0.01.

**Abbreviations**: Ctrl, control; Cal, calcitriol; Tacal, tacalcitol; OVX, ovariectomized.

Among CD3^+^CD4^+^ lymphocytes, we analyzed the population of IL-17-positive cells (Th17 lymphocytes). Tacalcitol increased Th17 lymphocytes in the lung tissue of young 4T1 tumor-bearing mice (Figure 4A). In aged OVX mice, the opposite trend was observed, with a significantly lower percentage of Th17 cells in tacalcitol-treated mice compared to those treated with calcitriol. Conversely, calcitriol increased the percentage of these cells in the tumor tissue of aged OVX 4T1 tumor-bearing mice (Figure 4B).

Analysis of other tissues in 4T1 and 67NR tumor-bearing mice showed no differences among treatment groups (Figure 4 and Supplementary Figure S4A–D). The only exception was the blood cell analysis of young 4T1 tumor-bearing mice, where tacalcitol increased the percentage of IL-17-positive cells compared to calcitriol (Supplementary Figure S4A).

### FoxP3 Expression in CD3^+^CD4^+^ Lymphocytes

CD3^+^CD4^+^ cells from tumors, blood, and selected organs were also analyzed for *Foxp3* expression (CD3^+^CD4^+^Foxp3^+^) (Supplementary Figure S5). In the tumor tissue of aged OVX 4T1 tumor-bearing mice, tacalcitol treatment increased the percentage of FoxP3^+^ cells (Supplementary Figure S5A). In lung tissue, calcitriol (*p* = 0.0535) and tacalcitol (*p* = 0.0688) showed a trend toward increasing the Treg population in young 4T1 tumor-bearing mice (Supplementary Figure S5B). The percentage of FoxP3^+^ splenocytes decreased in aged 4T1 tumor-bearing mice treated with tacalcitol (Supplementary Figure S5C). In addition to tumors, regional lymph nodes and blood from 4T1 tumor-bearing mice were analyzed (Supplementary Figure S5D and E). The only significant change was observed in the blood of aged mice, where calcitriol decreased the percentage of FoxP3^+^ cells (Supplementary Figure S5E).

### Characteristics of CD3^+^CD4^+^ Lymphocytes in Terms of Expression of OPN Receptors

CD3^+^CD4^+^ lymphocytes were analyzed for the expression of selected molecules described as OPN receptors, including three integrins (CD29, CD51, and CD61) and the CD44 receptor. In young mice bearing either 4T1 or 67NR tumors, the expression of these molecules was not significantly affected in various organs (Supplementary Figures S6A and S7A). The only observed change was an increase in CD44^+^ splenocytes in calcitriol-treated young 4T1 tumor-bearing mice (Supplementary Figure S6A). In aged 4T1 tumor-bearing mice, tacalcitol significantly decreased the percentage of CD29^+^ cells in tumor tissue, while calcitriol increased CD29^+^ cells in the blood (Supplementary Figure S6B). Tacalcitol increased the percentage of CD51^+^ cells in tumor tissue but decreased it in the spleen of aged 4T1 tumor-bearing mice. In the blood of these mice, calcitriol increased the percentage of CD44^+^ cells (Supplementary Figure S6B). In aged 67NR tumor-bearing mice, the only observed change was an increase in CD44^+^ cells in the spleen (Supplementary Figure S7B). Supplementary Figure S8 illustrates the gating strategy used for the OPN receptor expression analysis by flow cytometry.

### Selected Genes and Protein Expression in CD3^+^CD4^+^ Splenocytes

**Figure 5:**
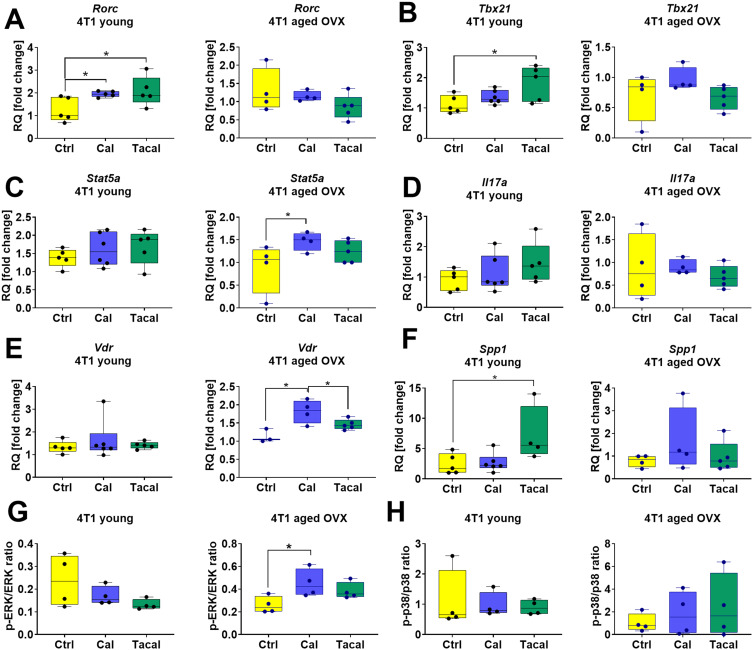
Selected genes and proteins expression in CD4^+^ T cells isolated from spleens of 4T1 tumor bearing young and aged OVX mice. (**A**) *Rorc*, (**B**) *Tbx21*, (**C**) *Stat5a*, (**D**) *Il17a*, (**E**) *Vdr*, (**F**) *Spp1*, (**G**) p-ERK/ERK ratio, (**H**) p-p38/p-38 ratio. (**A**–**F**) Analysis of gene expression was performed using specially designed TaqMan Array 96-Well FAST Plate Mouse FoxP3 plates coated with probes for the analysis of specific genes. Endogenous controls (*B2m, Actb*, and *Sdha*) were selected, and a comparative ΔΔCt analysis was performed. Samples from the group of control mice were selected as calibrators to obtain the RQ parameter. Analysis was performed using ExpressionSuite Software v1.3. (**G** and **H**) The Western blot analysis was performed in a Jess Simple Western System. The samples were normalized to total protein, and then the p-ERK/ERK or p-p38/p38 ratio was calculated. *N* = 4–6. Statistical analysis: (**A**–**F**) Sidak’s multiple comparisons test. (**G** and **H**) Tukey’s multiple comparisons test; *p < 0.05.

**Abbreviations**: *Il17a*, interleukin 17A; *Spp1*, osteopontin; *Rorc*, RAR related orphan receptor C; *Tbx21*, T-box transcription factor 21; *Vdr*, vitamin D receptor; *Stat5a*, signal transducer and activator of transcription 5A; Ctrl, control; Cal, calcitriol; Tacal, tacalcitol; OVX, ovariectomized.

**Figure 6:**
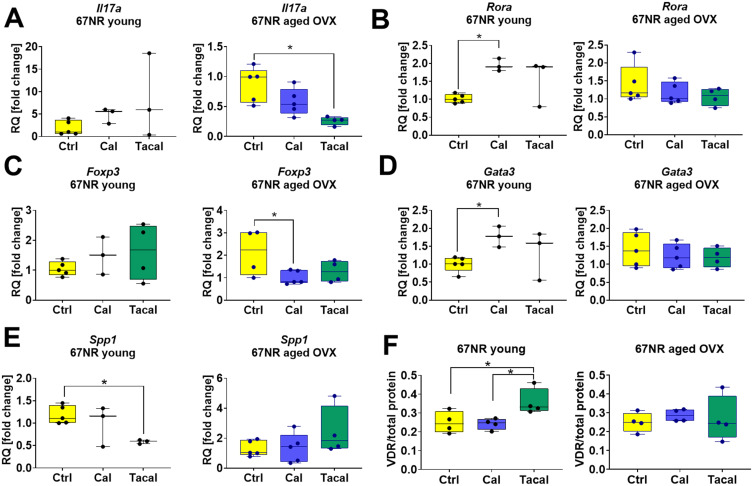
Selected genes and proteins expression in CD4^+^ T cells isolated from spleens of 67NR tumor bearing young and aged OVX mice. (**A**) *IL17a*, (**B**) *Rora*, (**C**) *Foxp3*, (**D**) *Gata3*, (**E**) *Spp1*, (**F**) VDR. (**A**–**E**) Analysis of gene expression was performed using specially designed TaqMan Array 96-Well FAST Plate Mouse FoxP3 plates coated with probes for the analysis of specific genes. Endogenous controls (*B2m* and *Actb*) were selected and a comparative ΔΔCt analysis was performed. Samples from the group of control mice were selected as calibrators to obtain the RQ parameter. Analysis was performed using ExpressionSuite Software v1.3. (**F**) The Western blot analysis was performed in a Jess Simple Western System. The samples were normalized to total protein. *N* = 3–5. Statistical analysis: Sidak’s multiple comparisons test; *p < 0.05.

**Abbreviations**: *Il17a*, interleukin 17A; *Spp1*, osteopontin; *Rora*, RAR related orphan receptor A; *Gata3*, GATA binding protein 3; *Foxp3*, forkhead box P3; VDR, vitamin D receptor; Ctrl, control; Cal, calcitriol; Tacal, tacalcitol.

Further characterization of CD3^+^CD4^+^ lymphocytes isolated from the spleen included the analysis of selected genes related to T lymphocyte subpopulation differentiation and the mechanisms of vitamin D_3_ action (Figures 5, 6, Supplementary Figures S9 and S10). In 4T1 tumor-bearing mice, calcitriol and tacalcitol significantly increased *Rorc* expression, while tacalcitol also increased *Tbx21* expression (Figure 5A and B). In aged mice, calcitriol increased *Stat5a* and *Vdr* expression (Figure 5C and E). Tacalcitol increased *Spp1* mRNA expression in young 4T1 tumor-bearing mice (Figure 5F).

In aged OVX 67NR tumor-bearing mice, tacalcitol decreased *Il17a* expression (Figure 6A), while calcitriol reduced *Foxp3* expression (Figure 6C). Increased *Rora* and *Gata3* expression was observed in young 67NR tumor-bearing mice (Figure 6B and D). *Spp1* expression was decreased by tacalcitol in young 67NR mice (Figure 6E). Analysis of phosphorylated ERK and p38 in CD3^+^CD4^+^ lymphocytes revealed an increase in ERK phosphorylation following calcitriol treatment in aged 4T1 tumor-bearing mice (Figure 5G–H). Additionally, tacalcitol increased VDR expression in young 67NR (Figure 6F) and aged 4T1 tumor-bearing mice (Supplementary Figure S11F). The expression levels of ERK, p-ERK, p38, and p-p38 are presented in Supplementary Figures S11 and S12.

### Differentiation of Th17 Lymphocytes in the Presence of CD29, CD51 and CD44 Blocking Antibodies

To evaluate functional responses, CD4^+^ splenocytes were differentiated into Th17 cells ex vivo under polarizing conditions, with additional blockade of OPN receptors (CD29, CD51, CD44).

**Figure 7:**
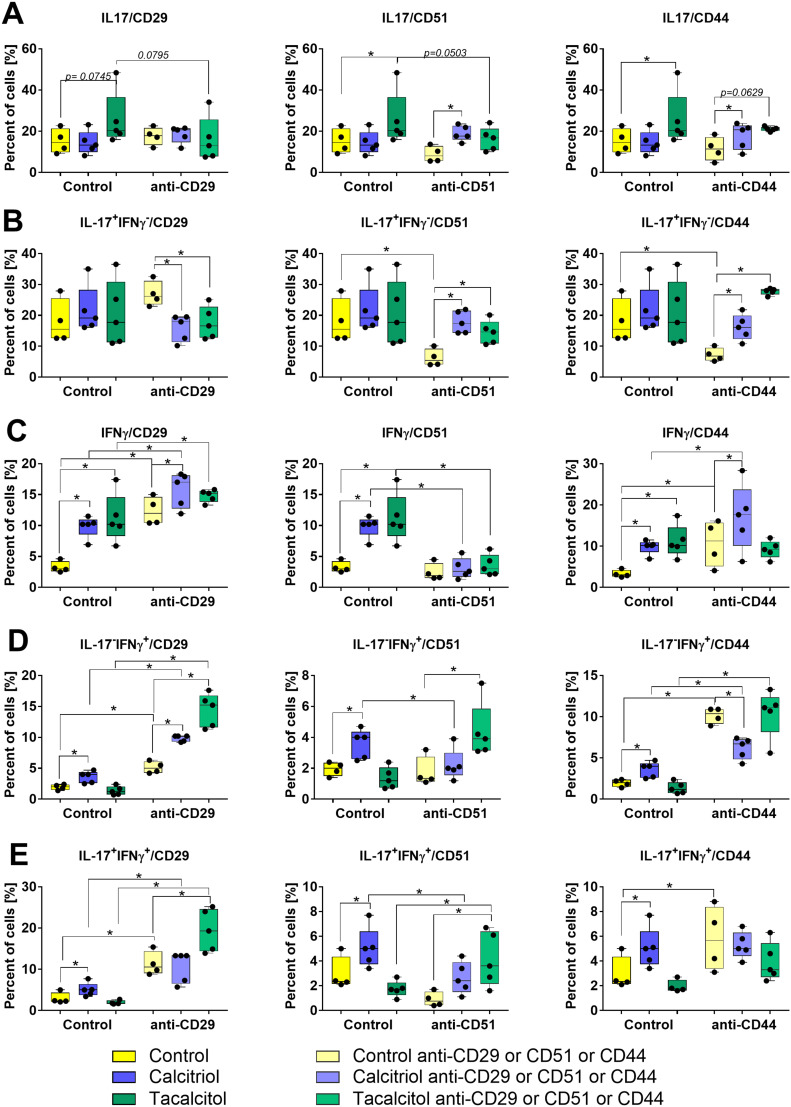
Characteristic of Th17 lymphocytes ex vivo differentiated with CD29, CD51, and CD44 blockade. Percentage of (**A**) all IL-17^+^, (**B**) IL-17^+^IFNγ^−^, (**C**) all IFNγ^+^, (**D**) IL-17^−^IFNγ^+^, (**E**) double positive IL-17^+^IFNγ^+^ cells among CD3^+^CD4^+^ splenocytes differentiated toward Th17 in control conditions (left part of each graph) and with blocking antibodies (right part of each graph). Left column of graphs: CD29 blocking, middle column: CD51 blocking, and right column, CD44 blocking. CD3^+^CD4^+^ splenocytes isolated from 4T1 young mice treated with calcitriol and tacalcitol (blocked or not with antiCD29, antiCD51, and antiCD44 antibodies before seeding) were differentiated ex vivo on plates coated with antiCD3 and antiCD28 antibodies with the presence of IL-6 and TGF-β. On the last day, cells were incubated with PMA (50 ng/mL) + ionomycin (500 ng/mL) + brefeldin A (5 µg/mL) and incubated for 4 h. The cells were analyzed by flow cytometry for intracellular expression of IL-17 and IFNγ. *N* = 4–5. Statistical analysis: Sidak’s multiple comparisons test; *p<0.05.

**Abbreviations**: IL-17, interleukin 17; IFNγ, interferon γ.

CD3^+^CD4^+^ splenocytes isolated from young 4T1 tumor-bearing mice were differentiated into Th17 lymphocytes. In tacalcitol-treated young mice, the percentage of lymphocytes expressing IL-17 was significantly higher (Figure 7A, control group); however, this effect was not observed when IFNγ^+^ cells were excluded from the analysis (Figure 7B). In mice treated with calcitriol and tacalcitol, an increased percentage of IFNγ^+^ cells among CD3^+^CD4^+^ splenocytes was noted (Figure 7C). When IL-17^+^ cells were excluded, a significant increase in IFNγ^+^ cells was observed only after calcitriol treatment (Figure 7D). Additionally, the percentage of IL-17^+^IFNγ^+^ double-positive cells increased in splenocytes from calcitriol-treated young mice during Th17 differentiation (Figure 7E).

**Figure 8:**
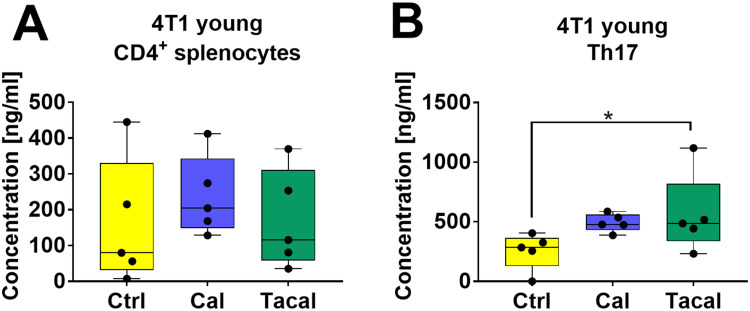
Osteopontin level in culture media of CD4^+^ splenocytes and induces Th17 cells from young mice bearing 4T1 mouse breast cancer. (**A**) CD4^+^ splenocytes; (**B**) CD4^+^ splenocytes differentiated ex vivo in Th17 differentiation condition. *N* = 5. Statistical analysis: Sidak’s multiple comparison test; *p<0.05.

**Abbreviations**: Ctrl, control; Cal, calcitriol; Tacal, tacalcitol.

During culture in the presence of Th17 differentiation factors, blocking antibodies against CD29, CD51, and CD44 were used. Blocking CD29 did not affect IL-17 expression in control mice but inhibited tacalcitol-induced stimulation of IL-17^+^ cells (Figure 7A, left graph). Furthermore, when the IL-17^+^IFNγ^−^ cell population was analyzed under CD29 blockade in cells from both calcitriol- and tacalcitol-treated mice, a significant decrease in the percentage of these cells was observed (Figure 7B, left graph). Blocking CD29 also led to an increased percentage of IFNγ^+^ cells across all groups of mice (Figures 7C and 8C). However, the increased percentage of IFNγ^+^ lymphocytes as an effect of calcitriol and tacalcitol treatment was preserved despite CD29 blockade. IL-17^−^IFNγ^+^ cells with blocked CD29 generally behaved similarly to the entire IFNγ^+^ population, however, the effect of stimulating IFNγ expression by calcitriol and tacalcitol was more pronounced (Figure 7D). The pattern of IL-17^+^IFNγ^+^ double-positive cells after CD29 blockade was similar to that observed in other IFNγ-expressing cell subpopulations (Figure 7E).

Blocking CD51 did not significantly affect Th17 differentiation in young mice but led to an increase in IL-17^+^ splenocytes from calcitriol-treated mice compared to controls (Figure 7A, middle graph). Analysis of the IL-17^+^IFNγ^−^ cell subpopulation showed a decreased level of these cells in the CD51-blocked control group compared to non-blocked control cells. However, IL-17^+^IFNγ^−^ cells were stimulated following CD51 blocking when derived from both calcitriol- and tacalcitol-treated mice (Figure 7B, middle graph). Conversely, after CD51 blocking, IFNγ stimulation was inhibited in cells from calcitriol- and tacalcitol-treated mice (Figure 7C). This inhibition was confirmed for calcitriol treatment in IL-17^−^IFNγ^+^ cells. Under CD51-blocking conditions, tacalcitol stimulated IL-17^+^IFNγ^−^ lymphocytes (Figure 7D). The pattern of IL-17^+^IFNγ^+^ double-positive cells after CD51 blocking was similar to that of the IL-17^−^IFNγ^+^ subpopulation (Figure 7E).

Blocking CD44 did not significantly affect the percentage of IL-17^+^ control cells (Figure 7A, right graph). However, IL-17^+^IFNγ^−^ cells decreased in the CD44-blocked control group compared to non-blocked controls, while all IFNγ^+^ populations increased (Figure 7B). The stimulatory effect of calcitriol and tacalcitol under CD44-blocking conditions was observed for the IL-17^+^IFNγ^−^ cell population, similar to the IL-17^+^ cells (Figure 7A and B). In contrast to the overall IFNγ^+^ population, calcitriol decreased IL-17^−^IFNγ^+^ cells under CD44-blocking conditions (Figure 7D). The pattern of IL-17^+^IFNγ^+^ double-positive cells after CD44 blocking was similar to that of the IL-17^−^IFNγ^+^ subpopulation (Figure 7E). Supplementary Figure S13 presents the gating scheme for IL-17 and IFNγ intracellular analysis by flow cytometry.

### OPN Level in Culture Supernatants

Supernatants from CD4^+^ splenocyte cultures and induced Th17 lymphocytes from young 4T1 tumor-bearing mice were analyzed for OPN secretion (Figure 8A and B). An increased concentration of OPN was observed in the culture of induced Th17 cells derived from tacalcitol-treated mice (Figure 8B).

## Discussion

Our previous studies have shown that calcitriol and its analogs (tacalcitol, PRI-2205) exert differential effects on the metastatic progression of 4T1 breast cancer depending on the age of the mice.^21^,^23^ We have attributed this to the immunomodulatory activity of vitamin D₃ derivatives.^22^,^35^ It is well established that aging alters immune system function;^36^ for example, dendritic cells—which are essential for Th17 cell maturation—may lose or diminish their activity with age.^37^ Moreover, in our earlier work, we have demonstrated that vitamin D₃ compounds can modulate immune cell populations differently in young versus aged mice, both in healthy individuals^38^ and in those bearing tumors.^7^,^24^,^35^ The age-related hormonal changes (eg, ovariectomy in aged mice) also impact the outcome of VD_3_-modulated immune responses.^24^ These findings suggest that age-related changes in the immune microenvironment significantly influence the response to vitamin D₃-based treatments.

Previous studies demonstrated that calcitriol can stimulate blood flow in 4T1 mouse mammary gland tumors at a late stage of tumor development (days 21–24 after cell implantation).^21^ However, no changes in blood flow were observed in young mice bearing 67NR tumors at that time following calcitriol treatment.^10^ In the present study, we assessed blood flow in tumors at an early stage of development, revealing increased tumor blood flow and blood flow abnormalities in young mice bearing both metastatic 4T1 and nonmetastatic 67NR tumors. In addition, in young mice bearing 67NR tumors, these blood flow abnormalities were accompanied by a calcitriol-induced increase in vascular density, as well as an increase in pro-angiogenic VEGF plasma levels following both calcitriol and tacalcitol treatment. Interestingly, in aged mice, the effect on blood flow in 67NR tumors was the opposite, whereas no significant impact of the tested compounds was observed in 4T1 tumors, either at this early stage or in advanced tumors.^23^

Previously, we also demonstrated that calcitriol, tacalcitol, or a cholecalciferol-rich diet enhanced 4T1 lung and bone marrow metastasis in young mice^7^,^21^,^39^ while reducing this process in aged OVX mice.^23^ Here, in addition to the lungs, we confirmed these previously reported findings by showing pro-metastatic effects in young mice and antimetastatic effects in aged OVX mice bearing 4T1 tumors in the liver.

We previously linked these changes in blood flow and metastasis to an increased expression of genes encoding transcription factors associated with Th17 differentiation in IL-6 and TGF-β-stimulated splenocytes from 4T1-bearing, tacalcitol-treated young mice. This effect was absent in aged mice.^24^ Furthermore, calcitriol is known to regulate the expression of the *OPN* (*Spp1*) gene.^40^ In our study, we observed a modulatory effect of calcitriol and its analogs on *Spp1* expression. Specifically, in lung tissue from aged OVX mice bearing 4T1 tumors, *Spp1* expression was decreased by calcitriol analogs on day 28 of the experiment,^35^ whereas an increase in *Spp1* expression was observed in young mice.^22^

In the present study, we analyzed IL-17 expression in CD3^+^CD4^+^ T cells across different tissues in mice bearing both metastatic and nonmetastatic tumors. We also examined the expression of various molecules known as OPN receptors in these cells. Interestingly, tacalcitol increased the percentage of CD3^+^CD4^+^IL-17^+^ cells in the lungs of young 4T1 tumor-bearing mice while decreasing their percentage in aged OVX mice, aligning with the observed lung *Spp1* expression.^22^,^35^ IL-17, through NF-κB-mediated expression of MMP-2 and MMP-9, is a key driver of breast cancer invasiveness and metastasis.^41^,^42^ Furthermore, OPN is known to promote Th17 lymphocytes and inflammation.^28^,^43^

**Figure 9:**
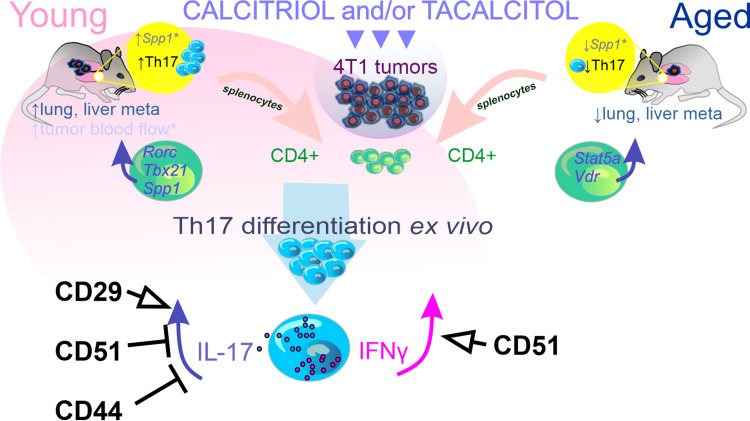
Calcitriol/tacalcitol stimulated tumor metastasis and blood flow in young mice bearing 4T1 murine breast cancer via impact on Th17 cells. The role of osteopontin receptors in this process is highlighted.

**Note**: *Published previously.^21^,^22^

**Abbreviations**: IL-17, interleukin 17; IFNγ, interferon γ; *Spp1*, osteopontin; *Rorc*, RAR-related orphan receptor C; *Tbx21*, T-box transcription factor 21; *Vdr*, vitamin D receptor; *Stat5a*, signal transducer and activator of transcription 5A.

In experimental autoimmune encephalomyelitis (EAE), inhibition of Th17 cells following calcitriol treatment has been reported in young mice.^19^ Additionally, calcitriol has been shown to inhibit Th17 cell generation in vitro.^44^ However, in our study, CD3^+^CD4^+^ splenocytes from young 4T1 tumor-bearing mice treated with tacalcitol (or both compounds) exhibited increased *Rorc, Tbx21*, and *Spp1* mRNA expression. This may lead to the fact that they are more likely to differentiate to Th17 and produce IFNγ under Th17 differentiation conditions (Figure 9).

The effects of calcitriol on CD3^+^CD4^+^ lymphocytes varied depending on mouse age and tumor type. Specifically, CD3^+^CD4^+^ splenocytes from aged OVX 4T1 tumor-bearing mice treated with calcitriol showed increased *Stat5a* and *Vdr* mRNA expression. Additionally, calcitriol stimulated p-ERK expression in CD3^+^CD4^+^ lymphocytes from these mice. *Stat5a*, a transcription factor essential for Th2 and Treg cell differentiation but not for Th17 or Th1 differentiation,^45^ may explain the lack of an effect of calcitriol or tacalcitol on IL-17 expression in splenocytes. In young 67NR tumor-bearing mice, calcitriol increased *Rora* and *Gata3* expression, while tacalcitol decreased *Spp1*. Conversely, in aged OVX 67NR tumor-bearing mice, tacalcitol reduced *Il17a* expression, and calcitriol decreased *Foxp3*. These opposing effects may contribute to the absence of significant differences in the CD3^+^CD4^+^ cell phenotype (IL-17 expression) in 67NR tumor-bearing young or aged mice.

IFNγ- and IL-17-co-expressing T cells have been documented in various human inflammatory autoimmune diseases.^46–48^ A Th17-derived Th1 subset has also been identified within tumors, showing a positive correlation with the survival status of cancer patients.^49^ Additionally, Muranski et al showed that Th17-polarized cells mediated the antitumor effect against advanced B16 melanoma in an IFNγ-dependent manner.^50^ In our study, splenocytes differentiated toward Th17, harvested from young mice treated with calcitriol and/or tacalcitol, exhibited increased expression of both IL-17 and IFNγ and produced higher levels of OPN. Moreover, calcitriol increased also double positive T lymphocytes percentage.

In further studies aiming to explain the mechanism that may be responsible for the increasing effect of calcitriol and tacalcitol in young mice bearing 4T1 tumors on IL-17 and IFNγ expression during ex vivo Th17 differentiation, we used blocking antibodies targeting selected integrins and the CD44 molecule. Previous studies have shown that CD44 blockade, and to a lesser extent blockade of β1 integrin subunit (CD29), significantly reduced Th17 differentiation, whereas blocking CD51 (αv) had no such effect, indicating that OPN exerts its effects on Th17 differentiation primarily through CD44 and CD29.^51^ Other studies have highlighted the role of CD61 (β3) as an OPN receptor involved in Th17 differentiation.^29^

CD29, CD51, and CD44 were selected for these studies because their expression on CD4^+^ cells from various organs was modulated to some extent by calcitriol and/or tacalcitol. Additionally, all three receptors have been implicated in Th17 differentiation.^43^,^51–53^ Among the CD29 subunits, α4β1, and α5β1 are the predominant integrins expressed on T lymphocytes.^54^,^55^ Furthermore, blocking CD29 or CD44 in CD4^+^CD45RA^−^CD25^+^ T cells stimulated with OPN, from acute coronary syndrome patients significantly decreased Th17 differentiation.^43^

Our results suggest that calcitriol and tacalcitol may regulate Th17 differentiation in 4T1 tumor-bearing mice through CD29. Specifically, CD29 signaling appears to be responsible for stimulating Th17 (but not Th1) differentiation in response to tacalcitol. In contrast, CD51 and CD44 may counteract the pro-differentiation effects of calcitriol on Th17 cells. Additionally, CD51 signaling appears to be important for calcitriol- and/or tacalcitol-induced IFNγ (Th1) stimulation under Th17 differentiation conditions.

CD44 signaling appears to play a distinct role in the effects of calcitriol and tacalcitol on IFNγ^+^ cells. In young mice, blocking CD44 did not alter the stimulatory effect of calcitriol on all IFNγ-expressing cells. However, in IL-17^−^IFNγ^+^ cells, CD44 blockade reversed the effect of calcitriol from stimulation to inhibition. Tacalcitol, which stimulated only the total IFNγ^+^ cell population, lost this ability when CD44 was blocked, suggesting that CD44 plays a role in tacalcitol’s Th1 pro-differentiation activity.

Interestingly, in our previous studies, we have shown increased expression of CD44, *Il23*, and *Irf4* (IL-23, IRF4, Th17 immune response drivers^56^) in tumor-associated macrophages (TAMs) isolated from 4T1 tumors growing in young mice and treated with calcitriol, alongside elevated OPN levels in tumor tissue. Moreover, these TAMs exhibited M2 macrophage characteristics. Additionally, 4T1 cells expressed high levels of COX-2 and released PGE_2_ following in vitro calcitriol treatment.^7^ Other studies using the 4T1 cancer model have shown that tumor-released PGE_2_ induces IL-23 production in the tumor microenvironment, causing Th17 cell expansion.^57^ Both IL-17 and PGE_2_ may, in turn, stimulate M2 macrophage polarization.^58^

These multidirectional effects of VD_3_ in young 4T1 tumor-bearing mice make tumor-conducing microenvironment leading to increased metastatic potential.

## Conclusion

Our study demonstrates that calcitriol and tacalcitol modulate Th17 cell differentiation and tumor progression in an age-dependent manner in murine models of breast cancer. These effects are linked to the expression and activity of OPN receptors on CD4^+^ T cells, particularly CD29, CD44, and CD51. We found that in young mice, CD29 contributed to the increased IL-17 expression in ex vivo differentiated Th17 cells derived from tacalcitol-treated mice, while CD51 and CD44 appeared to play opposing roles. Furthermore, IFNγ production in ex vivo differentiated Th17 cells from both calcitriol- and tacalcitol-treated young mice was mediated through CD51 integrin.

Taken together, our findings suggest that the balance between IL-17 and IFNγ in Th17 cells, shaped by vitamin D_3_ signaling and OPN receptor engagement, may determine whether Th17 cells exert pro- or anti-tumor effects.

## References

[1] Charoenngam N, Holick MF. Immunologic effects of vitamin D on human health and disease. *Nutrients*. 2020;12(7):1–28. doi:10.3390/NU12072097 PMC7400911 32679784

[2] El-Sharkawy A, Malki A. Vitamin D signaling in inflammation and cancer: molecular mechanisms and therapeutic implications. *Molecules*. 2020;25(14). doi:10.3390/MOLECULES25143219 PMC7397283 32679655

[3] Swami S, Krishnan A, Wang J, et al. Dietary vitamin D₃ and 1,25-dihydroxyvitamin D3 (calcitriol) exhibit equivalent anticancer activity in mouse xenograft models of breast and prostate cancer. *Endocrinology*. 2012;153(6):2576–2587. doi:10.1210/en.2011-1600 22454149 PMC3359605

[4] Swami S, Krishnan AV, Wang JY, et al. Inhibitory effects of calcitriol on the growth of MCF-7 breast cancer xenografts in nude mice: selective modulation of aromatase expression in vivo. *Horm Cancer*. 2011;2(3):190–202. doi:10.1007/s12672-011-0073-7 21686077 PMC3114631

[5] Marcinkowska E, Wallace GR, Brown G. The use of 1α,25-dihydroxyvitamin D3 as an anticancer agent. *Int J Mol Sci*. 2016;17(5). doi:10.3390/ijms17050729 PMC4881551 27187375

[6] Zhao C-N, Li Y, Meng X, et al. Insight into the roles of vitamins C and D against cancer: myth or truth? *Cancer Lett*. 2018;431:161–170. doi:10.1016/j.canlet.2018.05.039 29857128

[7] Stachowicz-Suhs M, Łabędź N, Anisiewicz A, et al. Calcitriol promotes M2 polarization of tumor-associated macrophages in 4T1 mouse mammary gland cancer via the induction of proinflammatory cytokines. *Sci Rep*. 2024;14(1):3778. doi:10.1038/s41598-024-54433-x 38355711 PMC10866890

[8] Łabędź N, Anisiewicz A, Stachowicz-Suhs M, et al. Dual effect of vitamin D3 on breast cancer-associated fibroblasts. *BMC Cancer*. 2024;24(1):209. doi:10.1186/s12885-024-11961-z 38360633 PMC10868064

[9] Stachowicz-Suhs M, Łabędź N, Milczarek M, et al. Vitamin D3 reduces the expression of M1 and M2 macrophage markers in breast cancer patients. *Sci Rep*. 2024;14(1):22126. doi:10.1038/s41598-024-73152-x 39333342 PMC11437092

[10] Łabędź N, Stachowicz-Suhs M, Psurski M, et al. Modulation of fibroblast activity via vitamin D3 is dependent on tumor type—studies on mouse mammary gland cancer. *Cancers*. 2022;14(19):4585. doi:10.3390/cancers14194585 36230508 PMC9559296

[11] Liu W, Zhang L, Xu H-J, et al. The anti-inflammatory effects of vitamin D in tumorigenesis. *Int J Mol Sci*. 2018;19(9):2736. doi:10.3390/ijms19092736 30216977 PMC6164284

[12] Adorini L, Daniel K, Penna G. Vitamin D receptor agonists, cancer and the immune system: an intricate relationship. *Curr Top Med Chem*. 2006;6(12):1297–1301. doi:10.2174/156802606777864890 16848743

[13] Cao Y, Du Y, Liu F, et al. Vitamin D aggravates breast cancer by inducing immunosuppression in the tumor bearing mouse. *Immunotherapy*. 2018;10(7):555–566. doi:10.2217/imt-2017-0131 29852828

[14] Martens PJ, Gysemans C, Verstuyf A, Mathieu C. Vitamin D’s effect on immune function. *Nutrients*. 2020;12(5). doi:10.3390/NU12051248 PMC7281985 32353972

[15] Hayes CE, Hubler SL, Moore JR, Barta LE, Praska CE, Nashold FE. Vitamin D actions on CD4+ T cells in autoimmune disease. *Front Immunol*. 2015;6(March):1–22. doi:10.3389/fimmu.2015.00100 25852682 PMC4364365

[16] Huang P, He XY, Xu M. Correlation of vitamin D3 with the expression of RORγt and Foxp3 mRNAs in the peripheral blood of myasthenia gravis patients. *Neuroimmunomodulation*. 2020;27(2):97–103. doi:10.1159/000510861 33271562

[17] Slominski AT, Kim TK, Li W, Yi AK, Postlethwaite A, Tuckey RC. The role of CYP11A1 in the production of vitamin D metabolites and their role in the regulation of epidermal functions. *J Steroid Biochem Mol Biol*. 2014;144:28–39. doi:10.1016/J.JSBMB.2013.10.012 24176765 PMC4002668

[18] Thangamani S, Kim M, Son Y, et al. Cutting edge: progesterone directly upregulates vitamin D receptor gene expression for efficient regulation of T cells by calcitriol. *J Immunol*. 2015;194(3):883–886. doi:10.4049/jimmunol.1401923 25548222 PMC4356636

[19] Joshi S, Pantalena L-C, Liu XK, et al. 1,25-dihydroxyvitamin D3 ameliorates Th17 autoimmunity via transcriptional modulation of interleukin-17A. *Mol Cell Biol*. 2011;31(17):3653–3669. doi:10.1128/MCB.05020-11 21746882 PMC3165548

[20] Slominski AT, Kim TK, Hobrath JV, et al. Endogenously produced nonclassical vitamin D hydroxy-metabolites act as “biased” agonists on VDR and inverse agonists on RORα and RORγ. *J Steroid Biochem Mol Biol*. 2017;173:42–56. doi:10.1016/j.jsbmb.2016.09.024 27693422 PMC5373926

[21] Anisiewicz A, Pawlik A, Filip-Psurska B, et al. Unfavorable effect of calcitriol and its low-calcemic analogs on metastasis of 4T1 mouse mammary gland cancer. *Int J Oncol*. 2017;52(1):103–126. doi:10.3892/ijo.2017.4185 29115583 PMC5743363

[22] Pawlik A, Anisiewicz A, Filip-Psurska B, et al. Calcitriol and its analogs establish the immunosuppressive microenvironment that drives metastasis in 4T1 mouse mammary gland cancer. *Int J Mol Sci*. 2018;19(7):2116. doi:10.3390/IJMS19072116 30037009 PMC6073894

[23] Anisiewicz A, Filip-Psurska B, Pawlik A, et al. Calcitriol analogues decrease lung metastasis but impair bone metabolism in aged ovariectomized mice bearing 4T1 mammary gland tumours. *Aging Dis*. 2019;10(5):977. doi:10.14336/AD.2018.0921 31595196 PMC6764735

[24] Pawlik A, Anisiewicz A, Filip-Psurska B, et al. Divergent effect of tacalcitol (PRI-2191) on Th17 cells in 4T1 tumor bearing young and old ovariectomized mice. *Aging Dis*. 2020;11(2):241. doi:10.14336/AD.2019.0618 32257539 PMC7069462

[25] Song Y, Yang JM. Role of interleukin (IL)-17 and T-helper (Th)17 cells in cancer. *Biochem Biophys Res Commun*. 2017;493(1):1–8. doi:10.1016/j.bbrc.2017.08.109 28859982

[26] Gulubova M, Ananiev J, Ignatova M, Halacheva K. Pro-tumor and anti-tumor functions of IL-17 and of TH17 cells in tumor microenvironment. *Acta Medica Bulg*. 2016;43(2):68–79. doi:10.1515/amb-2016-0019

[27] Shan M, Yuan X, Song LZ, et al. Cigarette smoke induction of osteopontin (SPP1) mediates TH17 inflammation in human and experimental emphysema. *Sci Transl Med*. 2012;4(117):117ra9. doi:10.1126/scitranslmed.3003041 PMC3956594 22261033

[28] Murugaiyan G, Mittal A, Weiner HL. Increased osteopontin expression in dendritic cells amplifies IL-17 production by CD4+ T cells in experimental autoimmune encephalomyelitis and in multiple sclerosis. *J Immunol*. 2008;181(11):7480–7488. 19017937 10.4049/jimmunol.181.11.7480 PMC2653058

[29] Zhao Q, Cheng W, Xi Y, et al. IFN-β regulates Th17 differentiation partly through the inhibition of osteopontin in experimental autoimmune encephalomyelitis. *Mol Immunol*. 2018;93:20–30. doi:10.1016/j.molimm.2017.11.002 29127843

[30] Lau WL, Leaf EM, Hu MC, et al. Vitamin D receptor agonists increase klotho and osteopontin while decreasing aortic calcification in mice with chronic kidney disease fed a high phosphate diet. *Kidney Int*. 2012;82(12):1261–1270. doi:10.1038/ki.2012.322 22932118 PMC3511664

[31] Jeon S-M-M, Shin E-A-A. Exploring vitamin D metabolism and function in cancer. *Exp Mol Med*. 2018;50(4):20. doi:10.1038/s12276-018-0038-9 29657326 PMC5938036

[32] Kuczma M, Podolsky R, Garge N, et al. Foxp3-deficient regulatory T cells do not revert into conventional effector CD4+ T Cells but constitute a unique cell subset. *J Immunol*. 2009;183(6):3731–3741. doi:10.4049/jimmunol.0800601 19710455 PMC2771373

[33] Scirka B, Szurek E, Pietrzak M, et al. Anti-GITR antibody treatment increases TCR repertoire diversity of regulatory but not effector T cells engaged in the immune response against B16 melanoma. *Arch Immunol Ther Exp*. 2017;65(6):553–564. doi:10.1007/s00005-017-0479-1 PMC5688217 28638937

[34] DuPre’ SA, Hunter KW. Murine mammary carcinoma 4T1 induces a leukemoid reaction with splenomegaly: association with tumor-derived growth factors. *Exp Mol Pathol*. 2007;82(1):12–24. doi:10.1016/j.yexmp.2006.06.007 16919266

[35] Anisiewicz A, Pawlik A, Filip-Psurska B, Wietrzyk J. Differential impact of calcitriol and its analogs on tumor stroma in young and aged ovariectomized mice bearing 4T1 mammary gland cancer. *Int J Mol Sci*. 2020;21(17):6359. doi:10.3390/ijms21176359 32887237 PMC7503326

[36] Salminen A, Kaarniranta K, Kauppinen A. Immunosenescence: the potential role of myeloid-derived suppressor cells (MDSC) in age-related immune deficiency. *Cell Mol Life Sci*. 2019;76(10):1901–1918. doi:10.1007/s00018-019-03048-x 30788516 PMC6478639

[37] Agrawal A, Gupta S. Impact of aging on dendritic cell functions in humans. *Ageing Res Rev*. 2011;10(3):336–345. doi:10.1016/J.ARR.2010.06.004 20619360 PMC3030666

[38] Śnieżewska A, Anisiewicz A, Gdesz-Birula K, Wietrzyk J, Filip-Psurska B. Age-dependent effect of calcitriol on mouse regulatory T and B lymphocytes. *Nutr*. 2024;16(1):49. doi:10.3390/NU16010049 PMC10780377 38201878

[39] Anisiewicz A, Kowalski K, Banach J, et al. Vitamin d metabolite profile in cholecalciferol-or calcitriol-supplemented healthy and mammary gland tumor-bearing mice. *Nutrients*. 2020;12(11):1–28. doi:10.3390/nu12113416 PMC7695033 33172201

[40] Shen Q, Christakos S. The vitamin D receptor, Runx2, and the notch signaling pathway cooperate in the transcriptional regulation of osteopontin. *J Biol Chem*. 2005;280(49):40589–40598. doi:10.1074/jbc.M504166200 16195230

[41] Shibabaw T, Teferi B, Ayelign B. The role of Th-17 cells and IL-17 in the metastatic spread of breast cancer: as a means of prognosis and therapeutic target. *Front Immunol*. 2023;14. doi:10.3389/fimmu.2023.1094823 PMC10040566 36993955

[42] Roy L, Sahraei M, Schettini JL, Gruber HE, Besmer DM, Mukherjee P. Systemic neutralization of IL-17A significantly reduces breast cancer associated metastasis in arthritic mice by reducing CXCL12/SDF-1 expression in the metastatic niches. *BMC Cancer*. 2014;14(1):225. doi:10.1186/1471-2407-14-225 24674692 PMC3986611

[43] Zheng Y, Wang Z, Deng L, et al. Osteopontin promotes inflammation in patients with acute coronary syndrome through its activity on IL ‐17 producing cells. *Eur J Immunol*. 2012;42(10):2803–2814. doi:10.1002/eji.201242475 22711477

[44] Ikeda U, Wakita D, Ohkuri T, et al. 1α,25-dihydroxyvitamin D3 and all-trans retinoic acid synergistically inhibit the differentiation and expansion of Th17 cells. *Immunol Lett*. 2010;134(1):7–16. doi:10.1016/j.imlet.2010.07.002 20655952

[45] Lee J, Lozano-Ruiz B, Yang FM, Fan DD, Shen L, González-Navajas JM. The multifaceted role of Th1, Th9, and Th17 cells in immune checkpoint inhibition therapy. *Front Immunol*. 2021;12. doi:10.3389/fimmu.2021.625667 PMC7994325 33777008

[46] Kurschus FC, Croxford AL, P. Heinen A, Wörtge S, Ielo D, Waisman A. Genetic proof for the transient nature of the Th17 phenotype. *Eur J Immunol*. 2010;40(12):3336–3346. doi:10.1002/eji.201040755 21110317

[47] Harbour SN, Maynard CL, Zindl CL, Schoeb TR, Weaver CT. Th17 cells give rise to Th1 cells that are required for the pathogenesis of colitis. *Proc Natl Acad Sci U S A*. 2015;112(22):7061–7066. doi:10.1073/PNAS.1415675112 26038559 PMC4460486

[48] Ronchi F, Basso C, Preite S, et al. Experimental priming of encephalitogenic Th1/Th17 cells requires pertussis toxin-driven IL-1β production by myeloid cells. *Nat Commun*. 2016;7. doi:10.1038/NCOMMS11541 PMC4873938 27189410

[49] Lei X, Xiao R, Chen Z, et al. Augmenting antitumor efficacy of Th17-derived Th1 cells through IFN-γ-induced type I interferon response network via IRF7. *Proc Natl Acad Sci U S A*. 2024;121(47):e2412120121. doi:10.1073/PNAS.2412120121/SUPPL_FILE/PNAS.2412120121.SAPP.PDF 39541355 PMC11588128

[50] Muranski P, Boni A, Antony PA, et al. Tumor-specific Th17-polarized cells eradicate large established melanoma. *Blood*. 2008;112(2):362–373. doi:10.1182/blood-2007-11-120998 18354038 PMC2442746

[51] Chen G, Zhang X, Li R, et al. Role of osteopontin in synovial Th17 differentiation in rheumatoid arthritis. *Arthritis Rheum*. 2010;62(10):2900–2908. doi:10.1002/art.27603 20533542

[52] Guan H, Nagarkatti PS, Nagarkatti M. CD44 reciprocally regulates the differentiation of encephalitogenic Th1/Th17 and Th2/regulatory T Cells through epigenetic modulation involving DNA methylation of cytokine gene promoters, thereby controlling the development of experimental autoimmune encephalomyelitis. *J Immunol*. 2011;186(12):6955–6964. doi:10.4049/jimmunol.1004043 21551360 PMC3650091

[53] Su P, Chen S, Zheng YH, et al. Novel function of extracellular matrix protein 1 in suppressing Th17 cell development in experimental autoimmune encephalomyelitis. *J Immunol*. 2016;197(4):1054–1064. doi:10.4049/jimmunol.1502457 27316685 PMC4975973

[54] Iwata S, Ohashi Y, Kamiguchi K, Morimoto C. Beta 1-integrin-mediated cell signaling in T lymphocytes. *J Dermatol Sci*. 2000;23(2):75–86. doi:10.1016/S0923-1811(99)00096-1 10808124

[55] Puente LG, Ostergaard HL. Beta 1/beta 3 integrin ligation is uncoupled from ERK1/ERK2 activation in cytotoxic T lymphocytes. *J Leukoc Biol*. 2003;73(3):391–398. doi:10.1189/JLB.0402199 12629153

[56] Filip-Psurska B, Zachary H, Strzykalska A, Wietrzyk J. Vitamin D, Th17 lymphocytes, and breast cancer. *Cancers*. 2022;14(15):3649. doi:10.3390/cancers14153649 35954312 PMC9367508

[57] Qian X, Gu L, Ning H, et al. Increased Th17 cells in the tumor microenvironment is mediated by IL-23 via tumor-secreted prostaglandin E2. *J Immunol*. 2013;190(11):5894–5902. doi:10.4049/jimmunol.1203141 23645882 PMC3660540

[58] Liu L, Ge D, Ma L, et al. Interleukin-17 and prostaglandin E2 are involved in formation of an M2 macrophage-dominant microenvironment in lung cancer. *J Thorac Oncol*. 2012;7(7):1091–1100. doi:10.1097/JTO.0b013e3182542752 22534817 PMC3378786

